# The Inhibitory Effect of Perceived Organizational Politics on Employee Voice Behavior: A Moderated Mediation Model

**DOI:** 10.3389/fpsyg.2021.727893

**Published:** 2021-09-17

**Authors:** Qin Liu, Hao Zhou, Xinyi Sheng

**Affiliations:** Business School, Sichuan University, Chengdu, China

**Keywords:** voice behavior, perceived organizational politics, sense of impact, conservation of resources theory, organizational embeddedness

## Abstract

Research on the mediating mechanisms and boundary conditions of perceived organizational politics’ (POP) effect on employee voice is underdeveloped. Based on conservation of resources theory, we proposed a moderated mediation model in which organizational embeddedness acts as a mediator to explain why POP inhibits promotive and prohibitive voice. Additionally, we posited sense of impact as a boundary condition affecting this relationship. A time-lagged survey of 227 employed MBA students from a university in southwestern China revealed that organizational embeddedness mediates the relationship between POP and promotive and prohibitive voice, and sense of impact moderates the relationship between POP and promotive voice, such that the relationship is stronger when sense of impact is weaker. The moderating effect was not significant for prohibitive voice. These findings have implications for theory, practice, and further organizational research.

## Introduction

Employee voice behavior—defined as voluntarily expressing work-related ideas or concerns (Chamberlin et al., 2017; Liang, 2021)—can promote organizational function effectively in a dynamic environment (Crant et al., 2011; Morrison, 2011, 2014). It has been shown to be positively associated with many work outcomes at multiple levels, such as organizational learning, financial performance, innovation, effectiveness, and crisis prevention (Detert and Burris, 2007; Mackenzie et al., 2011; Morrison, 2014; Bashshur and Oc, 2015; Chamberlin et al., 2017); group performance, effectiveness, decision making, and creativity (Nemeth et al., 2001; Mackenzie et al., 2011; Detert et al., 2013); and individual performance, thriving, psychological well-being and career success (Seibert et al., 2001; Maynes and Podsakoff, 2014; Yousaf et al., 2019; Chen et al., 2020). Given the importance of voice, although important antecedents have been studied, scholars and practitioners still strive to understand the motivating mechanism of voice behavior to improve the effectiveness of organizational operations (Kong et al., 2017). This paper will verify how perceived organizational politics (POP), an inevitable organizational context, affects voice behavior.

POP refers to the perception of the degree of colleagues’ and supervisors’ self-interested behavior, which is pervasive in the workplace (Ferris et al., 1989). It has attracted increasing attention from scholars and practitioners in recent decades (Li et al., 2014; Ferris et al., 2019). Numerous studies have indicated that POP has a strong influence on work attitudes and behaviors, such as psychological strain, job satisfaction, turnover intentions, organizational citizenship behavior (OCB; Organ, 1988), and task performance (Chang et al., 2009; Al Jisr et al., 2020). Nevertheless, the study of POP’s effect on voice behavior is underdeveloped. Only a few studies (Li et al., 2014, 2020; Bergeron and Thompson, 2020), have examined this issue, demonstrating that POP negatively affects voice behavior.

Li et al. (2014, 2020) and Bergeron and Thompson (2020) have focused primarily on the risks associated with voice behavior, mainly regarding it as a consumption behavior. However, voice behavior not only consumes resources but also helps individuals obtain resources, such as improving their image and ratings (Van Dyne and LePine, 1998; Lin and Johnson, 2015). Therefore, it may be more appropriate to use conservation of resources theory (Hobfoll, 1989, 2001) to analyze voice behavior in the POP context, considering both the investment and consumption associated with voice behavior. Extant studies have demonstrated that psychological safety, psychological uncertainty, and perceived organizational support mediate the relationship between POP and voice behavior (Li et al., 2014, 2020; Bergeron and Thompson, 2020), but such variables cannot adequately explain the impact of POP on voice behavior. Moreover, organizational embeddedness encompasses aspects of work lives that can increase employees’ attachment to the organization, consisting of links, fit, and sacrifice (Singh et al., 2020). It reflects the state of the resource pool (Halbesleben and Wheeler, 2008; Harris et al., 2011) and is influenced mainly by the organizational and work context. Individuals with different resource pools will affect how they view voice behaviors, that is, whether they regard voice behavior as investment or consumption (Halbesleben et al., 2014; Hobfoll et al., 2018). Based on this logic, we posit organizational embeddedness as a mediating variable to explain the influence of POP on promotive and prohibitive voice, thus expanding the existing research on the POP-voice linkage.

**FIGURE 1:**
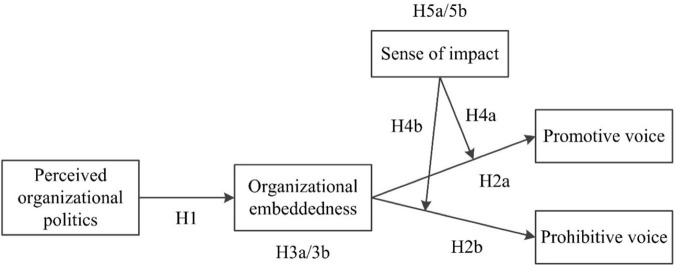
The conceptual model.

Additionally, COR theory holds that the initial resource pool (including internal and external resources) will affect employees’ choice of investment or conservation behaviors (Hobfoll, 1989; Halbesleben et al., 2014), organizational embeddedness is an external resource (Halbesleben and Wheeler, 2008; Lee et al., 2014), so this paper will introduce an internal resource to investigate how they affect voice behavior and how they relate to external resources. Individuals with different internal resources may exhibit differences in this respect. Employees with a strong sense of impact—referring to the extent to which employees perceive that they can determine important work outcomes (Spreitzer, 1995; Tangirala and Ramanujam, 2008)—possess more internal resources (Brouer et al., 2011), which can help them mitigate the perceived risk of voice and make a difference, thus reducing dependence on external resources (e.g., organizational embeddedness; Kiazad et al., 2015; Singh et al., 2020). Collectively, we tested sense of impact as a moderating factor, which weakens not only the relationship between organizational embeddedness and voice behavior but also the mediating effect of organizational embeddedness on the relationship between POP and voice behavior; further, we verified the substitutive relationship between internal and external resources. Figure 1 depicts our conceptual model.

Based on COR theory, our study investigates the indirect and conditional effects of POP on employee voice behavior and contributes to the theory and practice of organizational management. First, we use the resource perspective, operationalized as organizational embeddedness, as a mechanism to explain how POP inhibits employee voice behavior. Prior research has shown that POP is a stressor that results in many negative work consequences (Miller et al., 2008; Chang et al., 2009; Ferris et al., 2019), but the influence of POP on organizational embeddedness has not been explored sufficiently. In line with COR theory, our research explores how POP as a stressful social context affects employees’ resource pool (i.e., organizational embeddedness), and further affects voice behavior. Thus, from the COR perspective, we propose that employees’ resource pool (organizational embeddedness) transmits the impact of POP to voice behavior.

Second, this study identifies a boundary condition (i.e., sense of impact) under which the indirect effect of POP on voice behavior varies. Internal resources can help offset the negative effect of POP on voice behavior. Improving employees’ sense of impact, for instance, can thus motivate voice behavior.

Third, although COR theory holds that resources may be replaced, substituted, or in caravans (Hobfoll, 1989, 2001, 2011; Hobfoll et al., 2018), there are few empirical studies regarding these resources. To address this research gap and promote the effective use of resources in practice, this study empirically identifies the relationships among different types of resources (i.e., organizational embeddedness as an external resource and sense of impact as an internal resource) via the intermediary and conditional mechanism of POP-voice.

## Literature Review and Hypotheses

### Conservation of Resources Theory

COR theory contends that people strive to accumulate, protect, and retain valuable and limited resources (Hobfoll, 1989). At bottom, it is a stress theory, explaining when people feel stressed and how they respond to it (Hobfoll, 1989). Individuals strive to conserve current resources in stressful contexts and to accumulate new resources in non-stressful contexts (Hobfoll, 1989, 2001). This is the basic tenet of COR theory: protect existing resources (conservation) and obtain additional resources (acquisition; Hobfoll, 1989; Halbesleben et al., 2014). COR theory identifies four kinds of resources: objects, conditions, personal characteristics, and energies. Resources can also be classified as internal or external (Hobfoll, 1989). Internal resources refer to those “owned by employees themselves,” such as skills, optimism, and self-confidence. External resources refer to those “not owned by employees themselves,” such as social support and leadership (Hobfoll, 1989; Brouer et al., 2011; Fan et al., 2020). In this research, we conceptualize organizational embeddedness as an external resource and sense of impact as an internal resource.

### POP, Organizational Embeddedness, and Employee Voice

Hobfoll (1988) asserted that employees can accumulate resources from several kinds of support (e.g., high-quality leader-member exchange), and later research explored whether a stressful context (e.g., POP as a caravan passageway) hinders employees’ resource pools (Hobfoll, 2011). In an organizational context, organizational embeddedness reflects the state of the resource pool (Halbesleben and Wheeler, 2008; Harris et al., 2011; Ghosh et al., 2017). Further, COR theory posits that employees with larger resource pools have more opportunities to invest resources and can more readily resist the risk of loss, so they tend to invest to gain additional resources. On the contrary, employees with less resources are more defensive and inclined to protect existing resources (Halbesleben et al., 2014; Hobfoll et al., 2018). Thus, we tested whether POP weakens organizational embeddedness and influences promotive and prohibitive voice indirectly. To verify this argument, we must first demonstrate the relationship between POP and organizational embeddedness and then demonstrate the relationship between organizational embeddedness and promotive and prohibitive voice.

#### POP and Organizational Embeddedness

POP refers to employees’ subjective evaluation of the extent to which their colleagues and supervisors exhibit self-serving behaviors in the work environment (Ferris et al., 1989). It is prevalent in organizations and contributes considerably to employees’ stress (Ferris and Kacmar, 1992; Gilmore et al., 1996). Employees with strong POP believe that many political behaviors exist, regardless of the reality (Ferris et al., 1989). Organizational politics is ubiquitous and important in social contexts, affecting resource distribution by confusing the appraisal system, the reward system, and interpersonal relationships (Vigoda, 2001; Treadway et al., 2005; Li et al., 2020). Strong POP makes outcomes unpredictable, thus elevating the risk of losing resources due to unfair treatment and retaliation (Vigoda, 2001; Kimura, 2013).

Organizational embeddedness—which describes the resources (e.g., connections with colleagues, status, seniority) that cause an employee to adhere to the organization or the job (Halbesleben and Wheeler, 2008; Harris et al., 2011; Ghosh et al., 2017), helps us understand why employees stay in an organization, and predicts voluntary turnover intentions—is a sub-dimension of job embeddedness; another sub-dimension is community embeddedness (Mitchell et al., 2001). Compared with community embeddedness, organizational embeddedness can predict work-related behavior more accurately (Lee et al., 2004). Therefore, the current study focuses on organizational embeddedness, which includes three components: (1) **links**, the extent of connection with other people inside the organization; (2) **fit**, the extent of matching with the organization; and (3) **sacrifice**, the perceived material and psychological losses if one leaves the organization (Lee et al., 2014; Tan et al., 2019). Recent studies have identified embeddedness as a contextual resource (Kiazad et al., 2015; Singh et al., 2020) that is accumulated mostly from the organization and the work environment (Halbesleben and Wheeler, 2008; Lee et al., 2014). So, consistent with COR theory, we conceptualize it as an external resource.

Based on COR theory, in the stressful context of strong POP, employees are likely to protect existing resources (Hobfoll, 1989). In a high-POP environment, there are many self-serving “clubs” within the organization (Landells and Albrecht, 2017). Lacking trust in this self-interested, stressful environment (Bergeron and Thompson, 2020), employees will be more cautious to join a club to avoid belonging to the wrong club or becoming involved in competition or disputes between clubs (Khalid and Ahmed, 2016); they do this to avoid the potential loss of resources, which reduces the connection between members. Additionally, to adapt to or even utilize the coalition-based distribution environment, employees may join a “good” club to help them thrive in the organization and avoid backstabbing (Bedi and Schat, 2013; Landells and Albrecht, 2017). However, in such an environment, individuals are unlikely to join many clubs simultaneously. Collectively, employees prefer to “keep their noses clean” when the water is murky, which indicates fewer links between the organization and its members.

Moreover, in a high-POP environment, employees devote much time to cultivating interpersonal relationships (Vigoda, 2001; Erkutlu and Chafra, 2015), thus reducing time to perform job tasks properly, which leads them to feel unqualified (Lee et al., 2014; Hochwarter et al., 2020). Simultaneously, the self-interested behaviors induce many conflicts, suspicions, and jealous actions, leading to distrust and disrespect, which in turn results in diminished organizational fit (Tan et al., 2019). Additionally, efforts and returns are often unequal in a high-POP environment. Even if employees work hard in the organization, they may not receive their deserved returns, such as promotions or rewards (Vigoda, 2000; Li et al., 2014, 2020). Therefore, employees will lose relatively little resources—material or emotional—if they leave the organization. In other words, employees sacrifice less when they leave a high-POP organization.

Thus, in a high-POP work environment, most employees will have less links, fit, and sacrifice, which indicates a comparably lower resource pool (i.e., lower organizational embeddedness). For those few vested interests, in group members, they may have a short-lived strong internal connection, fit, and lost a lot of interest if they leave, but in a high-POP environment, this advantage may be fragile, unsustainable, and always afraid of being taken away by other alliances or in-group members. Therefore, in the long run, compared to low POP environment, individuals in high-POP environment still have limited links, fit, whether they are insiders or outsiders to the alliance. On the contrary, in a low-POP environment, the organization is fairer and employees trust each other (Ferris et al., 2002; Bedi and Schat, 2013); so, employees are willing to contact each other, and they perceive greater organizational inclusiveness. If they leave, they will lose much, such as fair treatment, trust, and respect. All these signify more links, better fit, and more sacrifice (i.e., high organizational embeddedness). In conclusion, we contend that POP inhibits the development of organizational embeddedness. Thus, we propose the following hypothesis:

Hypothesis 1: POP will be negatively associated with organizational embeddedness.

#### Organizational Embeddedness and Employee Voice

Voice behavior, first proposed by Hirschman (1970) in his exit-voice-loyalty model, ranges from “faint grumbling to violent protest.” Subsequent scholars extended the concept, contending that voice behavior is an extra-role behavior, which often challenges the status quo but aims to improve the organization through the expression of suggestions or concerns (Van Dyne et al., 2003, 2008). Then, based on previous studies, Liang et al. (2012) divided voice behavior into promotive voice and prohibitive voice according to its content, which has been widely applied by scholars (Qin et al., 2014; Kakkar et al., 2016; Song et al., 2019). On the one hand, the constructive attributes and positive motivation of voice behavior are conducive to employees improving their self-image and receiving high ratings (Van Dyne and LePine, 1998; Lin and Johnson, 2015). On the other hand, employees must consume time and cognitive resources to identify work-related ideas or problems and must use social skills to articulate them to others in the organization, which is usually costly and risky (Hochwarter et al., 2010; Kim et al., 2019; Song et al., 2019). In COR theory, the decision to voice ideas or concerns depends on a calculus of resource gains and losses (Tan et al., 2019; Huang et al., 2020; Li et al., 2020). Highly embedded employees will more frequently exhibit both promotive voice and prohibitive voice behaviors, but they do so for different reasons.

Promotive voice refers to employees’ expression of suggestions or ideas for improving the status quo of the work unit or organization (Liang et al., 2012). Promotive voice accompanies solutions, so good intentions will be recognized easily (Liang et al., 2012; Kong et al., 2017), and there will not be excessive risk. However, even if the risk associated with promotive voice is small, when the ideas cannot be adopted and make a difference, such behavior will be futile in attaining desired outcomes (e.g., good impressions, higher ratings, changes in the status quo), thus inhibiting employees’ motivation for promotive voice behavior (Hassan, 2015). However, highly embedded employees are more closely connected with leaders and colleagues and mesh better with organizational values and demands, facilitating employees’ deeper understanding of their work, generation of high-quality ideas or issues, receiving more support; thus, this paves the way for promotive voice and is conducive to the adoption of ideas (Kiazad et al., 2015; Singh et al., 2020). In other words, based on COR theory, highly embedded employees with strong resource pools (e.g., links with colleagues, fit with the organization) desire to employ an investment strategy (i.e., voice) to acquire new resources (Halbesleben et al., 2014). In contrast, weakly embedded employees with poor resource pools have less access to information and rarely receive others’ support. They tend to protect existing resources rather than engaging in resource-demanding behavior (i.e., voice).

Prohibitive voice refers to employees’ expression of concerns about existing or potential problems to prevent problems from becoming serious and to reduce losses (Van Dyne et al., 2008; Liang et al., 2012). It is a present-oriented challenge behavior (Van Dyne and LePine, 1998) and often damages the interests of stakeholders. This tends to impair interpersonal relations (Chamberlin et al., 2017; Ding et al., 2018), leading to many negative consequences (e.g., being retaliated against, being misunderstood as a troublemaker or complainer). These risks tend to discourage employees from speaking out about problems or mistakes (Morrison and Milliken, 2000). However, embeddedness resources (e.g., relationships with colleagues, fit with the organization) can help employees secure more trust and support, thus reducing the risk of being misunderstood or retaliated against. Thus, highly embedded employees with strong resource pools will employ more prohibitive voice behavior. On the contrary, weakly embedded employees (i.e., less relationships or fit) may be treated as complainers or troublemakers, although they have good intentions (Hassan, 2015; Faupel, 2020). Therefore, weakly embedded employees are reluctant to express their concerns so that they can avoid loss of resources, such as those caused by retaliation. Accordingly, we propose the following hypothesis:

Hypothesis 2: Organizational embeddedness will be positively associated with promotive voice (Hypothesis 2a) and prohibitive voice (Hypothesis 2b).

#### Organizational Embeddedness as a Mediator

As outlined above, COR theory suggests that embeddedness resources are limited when employees perceive strong organizational politics because such a distrustful, self-interested environment impairs relationships, fit, and sacrifice. In contrast, the perception of weak political behavior may enhance relationships, fit, and sacrifice because the organization is fair and employees trust and respect each other. In turn, higher organizational embeddedness stimulates employees to speak out (promotively and prohibitively), which is consistent with prior empirical studies (Ng and Feldman, 2013; Ng and Lucianetti, 2018; Tan et al., 2019). Accordingly, we propose the following hypothesis:

Hypothesis 3: POP will be indirectly and negatively associated with promotive voice behavior (Hypothesis 3a) and prohibitive voice behavior (Hypothesis 3b) through organizational embeddedness.

### The Moderating Roles of Sense of Impact

As argued above, POP impacts voice behavior through organizational embeddedness. Organizational embeddedness is a resource emanating from the environment (i.e., an external resource). POP’s impact may differ if we consider the resources controlled by employees themselves (i.e., internal resources), such as sense of impact. Therefore, from the resource perspective, we tested the moderating effect of sense of impact.

Impact refers to the extent to which an individual can influence strategic, administrative, or operating outcomes at work (Ashforth, 1989; Spreitzer, 1995). Moreover, sense of impact is a perception of that. When employees perceive they have a strong impact, they believe that their opinions are valued by their leaders and that they are capable of shaping and controlling their work contexts and outcomes (Tangirala and Ramanujam, 2012; Hassan, 2015; Wang et al., 2015). This confidence is a powerful psychological resource and increases employees’ sense of empowerment. Additionally, employees with a strong sense of impact generally believe that they should take more responsibility to improve the organization’s situation because they will enjoy more power, status, and respect (Fuller et al., 2006; Tangirala and Ramanujam, 2012). In sum, sense of impact is an important internal resource, which stems from employees’ ability and moral responsibility (Xanthopoulou et al., 2007) and can motivate employees to speak out (Hobfoll, 1989; Wang et al., 2015).

For employees with a strong sense of impact, there are abundant internal resources, which improves resource pools, as they comprise the sum of internal resources and external resources (Hobfoll, 1989; Fan et al., 2020). Based on COR theory, employees will exhibit more voice behavior when they have relatively strong resource pools (i.e., strong sense of impact). Furthermore, Hobfoll et al. (1990) argued that different resources might act as substitutes when they meet the same demand. With a relatively high level of resources (strong sense of impact), the marginal benefit produced by additional resources (organizational embeddedness) will diminish—that is, the positive effect of organizational embeddedness on voice behavior will weaken when employees have a strong sense of impact (Tan et al., 2019). In contrast, for employees with a weak sense of impact, due to limited internal resources, voice behavior depends more on external resources such as organizational embeddedness. Therefore, for these employees, organizational embeddedness has a stronger impact on voice behavior.

Specifically, there are some differences between promotive voice and prohibitive voice in terms of the role of sense of impact. Sense of impact helps convince employees that their opinions are valued and that they have an important influence on work decisions or outcomes (Brockner et al., 2004; Hassan, 2015). Accordingly, for promotive voice, internal resources can help enhance employees’ sense of responsibility for constructive change and help them secure more support for and adoption of their ideas (Fuller et al., 2006), which replaces some positive roles of organizational embeddedness in promotive voice behavior. Therefore, the positive relationship between organizational embeddedness and promotive voice will be weaker for employees with a strong sense of impact. For prohibitive voice, internal resources can help reduce employees’ perception of the risk associated with the behavior, which replaces some positive roles of organizational embeddedness in prohibitive voice behavior. Therefore, the positive relationship between organizational embeddedness and prohibitive voice will also be weaker for employees with a strong sense of impact. Synthesizing these insights, we propose the following hypothesis:

Hypothesis 4: Sense of impact will moderate the positive relationship between organizational embeddedness and promotive voice (Hypothesis 4a)/prohibitive voice (Hypothesis 4b), such that this relationship is stronger for employees with a weak sense of impact.

So far, we have delineated the mediating role of organizational embeddedness and the moderating role of sense of impact. Considering all our arguments, we contend that sense of impact moderates the indirect relationship between POP and voice behavior via organizational embeddedness. We thus propose the following hypothesis:

Hypothesis 5: Sense of impact will moderate the mediated relationship between POP and promotive voice (Hypothesis 5a)/prohibitive voice (Hypothesis 5b) through organizational embeddedness, such that this mediated relationship is stronger for employees with a weak sense of impact.

## Materials and Methods

### Participants and Procedure

We invited full-time employees from different industries and companies who were also registered as MBA students at a university in southwest China to complete the voluntary survey. We designed a three-wave measurement at intervals of 2 weeks to avoid common method bias, collecting the following information from participants: POP, sense of impact, demographics (gender, age, and organizational tenure), and zhongyong at Time 1; organizational embeddedness at Time 2; and promotive voice and prohibitive voice behavioral tendencies at Time 3.

Before distributing the questionnaire, we informed the participants of the survey procedure and assured them that their responses would remain anonymous. However, at each time that participants completed questionnaires, we asked them to leave their phone numbers, which would be used as a label to match the three-wave data and allow the participants to collect 10 yuan RMB in phone fee waivers as a reward for each questionnaire. To encourage participation for the full survey, we informed participants that if we successfully matched the three measurements, they would receive an additional 10 yuan RMB in phone fee waivers.

Of the 409 MBA students initially invited to participate in the survey at Time 1, 332 usable questionnaires (effectively, a response rate of 81.17%) remained after eliminating invalid questionnaires (i.e., those with missing data). At Time 2, we distributed 332 questionnaires to those who had participated in the first survey, and 274 usable surveys (effectively, a response rate of 82.53%) remained after matching responses and eliminating invalid questionnaires. At Time 3, we distributed 274 questionnaires to those who had participated in the previous two surveys, and 227 usable surveys (effectively, a response rate of 82.85%) remained after matching responses and eliminating invalid questionnaires. The full survey’s effective response rate was 55.50%. In our sample, 47.10% were female, the average age was 32.26 years (*SD* = 5.29), and the average organizational tenure was 6.08 years (*SD* = 4.95). Our sample originated from different enterprises, so there were no nested data.

### Measures

All scales used in this study were validated in previous studies. Except the three demographic items, we used a 5-point Likert scale for all survey items, which ranged from 1 (*strongly disagree*) to 5 (*strongly agree*). We presented all scales (except the Chinese measure of zhongyong) in Chinese following translation and back-translation procedures (Brislin, 1980). All the detailed scales are shown in Appendix.

#### Perceived Organizational Politics

We used the six-item POP scale developed and validated by Vigoda (2001). A sample item is “Favoritism rather than merit determines who gets ahead around here.” In this study, the Cronbach’s alpha value for the scale was 0.89.

#### Organizational Embeddedness

We used the three-item scale developed by Cunningham et al. (2005). A sample item is “I feel a strong link to my organization.” In this study, the Cronbach’s alpha value for the scale was 0.80.

#### Promotive Voice and Prohibitive Voice

We used the 10-item voice scale developed by Liang et al. (2012). Five items assess promotive voice (e.g., “I proactively develop and make suggestions for issues that may influence the unit”), and the other five items assess prohibitive voice (e.g., “I advise other colleagues against undesirable behaviors that would hamper job performance”). In this study, Cronbach’s alpha was 0.91 for the promotive voice subset and 0.90 for the prohibitive voice subset.

#### Sense of Impact

We measured sense of impact with Spreitzer’s (1995) three-item scale. In the real organizational situation, due to the differences in the professional fields of different departments, employees’ voice mostly occurs within department. Therefore, this study discusses and measures the employees’ sense of impact in their own department. A sample item is “My impact on what happens in my department is large.” In this study, the Cronbach’s alpha value for the scale was 0.86.

#### Control Variables

First, in line with previous voice research (Ng and Lucianetti, 2018; Li et al., 2020), we controlled for basic demographic variables, including gender, age, and organizational tenure. Second, given that (1) we collected data in the Chinese context and cultural factors may influence research conclusions, and (2) some studies have indicated that zhongyong—a value orientation, which advocates the adoption of a neutral and balance way on the basis of overall cognition to achieve the harmony between the individual and the environment (Du et al., 2014)—is associated with voice behavior (Duan and Ling, 2011; Qu et al., 2018), we controlled for zhongyong, which many scholars consider to be a typical Chinese cultural characteristic (Yang et al., 2016; Zhou et al., 2019). We used the six-item scale developed by Du et al. (2014) to measure zhongyong. A sample item is “I will find a compromise or balance between different opinions.” In this study, the Cronbach’s alpha value for the scale was 0.80.

### Analytical Approach

To establish the validity of the research constructs, we ran a series of confirmatory factor analyses (CFAs) using Mplus 8.0 (Muthén and Muthén, 2017). We evaluated model fit with indices including the root mean square error of approximation (RMSEA), the comparative fit index (CFI) and the Tucker-Lewis index (TLI). Then, in line with previous studies (e.g., Mo and Shi, 2018; Isaakyan et al., 2020), we used regression-based path analysis to test our hypotheses. Specifically, our study used a bootstrapping approach (5,000 iterations) to assess mediation and moderated mediation (Edwards and Lambert, 2007).

## Results

### Confirmatory Factor Analyses

We used chi-square difference tests to compare our hypothesized six-factor model to the alternative models that combined variables with high correlations. Table 1 shows the CFA results. The hypothesized six-factor model demonstrated reasonably good fit to the data (χ^2^ = 491.05, *df* = 335, CFI = 0.96, TLI = 0.95, RMSEA = 0.05), whereas the five-factor model (promotive voice and prohibitive voice combined), the four-factor model (POP and organizational embeddedness combined; promotive voice and prohibitive voice combined), and the one-factor model (all variables combined) fit poorly. This confirmed the distinctiveness of the six measures.

**TABLE 1:** Results of confirmatory factor analysis.

**Models**	**χ^2^**	**df**	**Δχ^2^ (Δdf)**	**RMSEA**	**CFI**	**TLI**
6-factor Model^a^(baseline)	491.05	335	–	0.05	0.96	0.95
5-factor Model^b^ (combining POP and OE)	721.14	340	230.09 (5)^***^	0.07	0.89	0.88
5-factor Model^c^ (combining OE and V1)	699.24	340	208.19 (5)^***^	0.07	0.90	0.89
5-factor Model^d^ (combining OE and V2)	729.91	340	238.86 (5)^***^	0.07	0.89	0.88
5-factor Model^e^ (combining V1 and V2)	935.07	340	444.02 (5)^***^	0.09	0.83	0.81
4-factor Model^f^ (combining POP and OE; combining V1 and V2)	1166.44	344	675.39 (9)^***^	0.10	0.76	0.74
1-factor Model^g^	2191.05	350	1700.00 (15)^***^	0.15	0.47	0.43

*POP, perceived organizational politics; OE, organizational embeddedness; V1, promotive voice; V2, prohibitive voice.*

*^a^Perceived organizational politics, zhongyong, organizational embeddedness, sense of impact, promotive voice, prohibitive voice.*

*^b^Combining perceived organizational politics and organizational embeddedness.*

*^c^Combining organizational embeddedness and promotive voice.*

*^d^Combining organizational embeddedness and prohibitive voice.*

*^e^Combining promotive voice and prohibitive voice.*

*^f^Combining perceived organizational politics and organizational embeddedness, Combining promotive voice and prohibitive voice.*

*^g^Combining all variables.*

*N = 227.*

^***^p < 0.001.

### Descriptive Statistics and Correlations

Table 2 presents the means, standard deviations, and correlations of the study variables. POP was negatively correlated with organizational embeddedness (*r* = −0.50, *p* < 0.001), promotive voice (*r* = −0.27, *p* < 0.001), and prohibitive voice (*r* = −0.35, *p* < 0.001). Organizational embeddedness was positively related to promotive voice (*r* = 0.54, *p* < 0.001) and prohibitive voice (*r* = 0.49, *p* < 0.001). Moreover, sense of impact was positively correlated with both promotive voice (*r* = 0.39, *p* < 0.001) and prohibitive voice (*r* = 0.37, *p* < 0.001).

**TABLE 2:** Descriptive statistics and correlations for study variables.

**Variables**	***M***	***SD***	**1**	**2**	**3**	**4**	**5**	**6**	**7**	**8**	**9**
1.Gender	1.47	0.50									
2.Age	32.26	5.29	−0.27***								
3.Tenure	6.08	4.95	−0.09	0.62***							
4.Zhongyong	4.09	0.47	0.07	0.05	0.13	(0.80)					
5.POP	2.53	0.88	−0.05	−0.15^*^	−0.00	−0.03	(0.89)				
6.OE	3.48	0.83	−0.05	0.21**	0.12	0.19**	−0.50***	(0.80)			
7.Sense of impact	3.42	0.79	−0.21**	0.30***	0.18**	0.19**	−0.22**	0.33***	(0.86)		
8.Promotive voice	3.92	0.60	0.00	0.17^*^	0.04	0.20**	−0.27***	0.54***	0.39***	(0.91)	
9.Prohibitive voice	3.48	0.75	−0.14^*^	0.32***	0.14^*^	0.09	−0.35***	0.49***	0.37***	0.52***	(0.90)

*N = 227. POP, perceived organizational politics; OE, organizational embeddedness, Cronbach’s alpha coefficients are on the diagonal in parentheses. Age and tenure measured in years. Gender 1 = male, 2 = female.*

*M, mean; SD, standard deviation.*

^*^p < 0.05, **p < 0.01, ***p < 0.001.

### Hypothesis Testing

**FIGURE 2:**
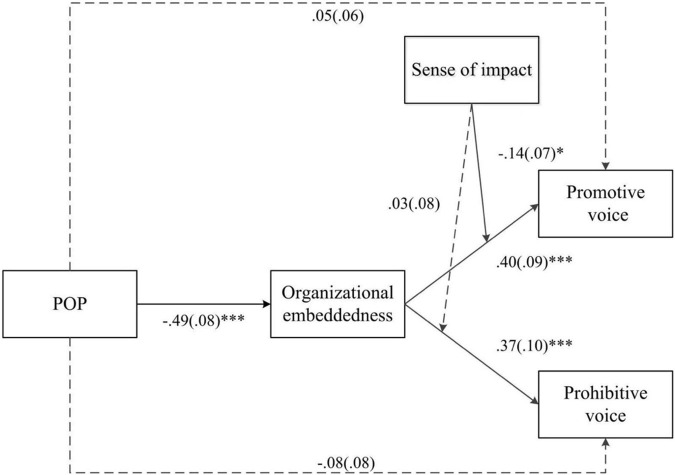
Path coefficients of the conceptual model. *N* = 227. Path coefficients and standard deviations from the conceptual model. For brevity, we did not present the effects of the control variables. Interested readers may contact the corresponding author for estimates of these effects. *^∗^p <* 0.05, ^∗∗∗^*p* < 0.001.

As shown in Figure 2, POP was negatively related to organizational embeddedness (*B* = −0.49, *p* < 0.001). Thus, Hypothesis 1 was supported. Further, organizational embeddedness was positively related to promotive voice (*B* = 0.40, *p* < 0.001) and prohibitive voice (*B* = 0.37, *p* < 0.001). This supported Hypotheses 2a and 2b.

Hypothesis 3 predicted that organizational embeddedness would mediate the relationship between POP and employee voice behavior. To estimate the hypothesized indirect relationship, we used a bootstrapping procedure (5,000 samples). The results indicated that there was a significant indirect relationship between POP and promotive voice via organizational embeddedness (indirect effect = −0.34, 95% bias-corrected bootstrap CI [−0.50, −0.22], excluding zero). Thus, Hypothesis 3a was supported. Additionally, the indirect relationship between POP and prohibitive voice via organizational embeddedness was significant (indirect effect = −0.23, 95% bias-corrected bootstrap CI [−0.37, −0.12], excluding zero). Thus, Hypothesis 3b was supported, as well.

**FIGURE 3:**
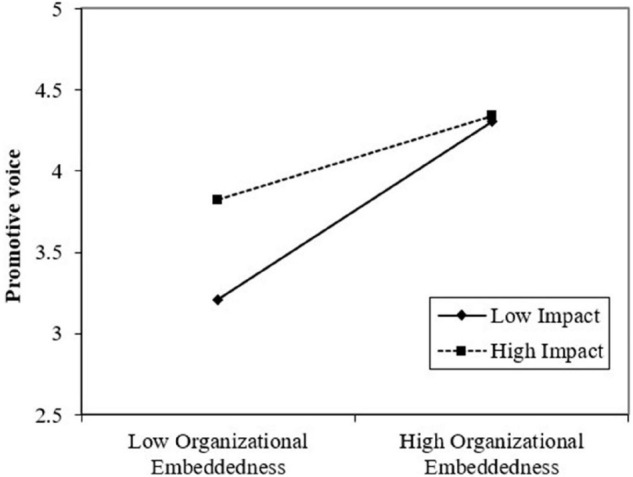
The moderating role of sense of impact for the relationship between organizational embeddedness and promotive voice.

Hypothesis 4 proposed sense of impact as a moderator of the relationship between organizational embeddedness and employee voice. Figure 2 shows that the interaction between organizational embeddedness and sense of impact significantly affected promotive voice (*B* = −0.14, *p* < 0.05) but did not significantly affect prohibitive voice (*B* = 0.03, *p* = 0.73). As Figure 3 illustrates, the positive relationship between organizational embeddedness and promotive voice was stronger for employees with a weak (*M*− 1 *SD*) sense of impact than for those with a strong (*M* + 1 *SD*) sense of impact. Thus, Hypothesis 4a was supported, but Hypothesis 4b was not supported.

Hypothesis 5 stated that the mediating effect of POP on employee voice through organizational embeddedness would be moderated by sense of impact. As shown in Table 3, in our analysis with 5,000 bootstrap samples, there was a significant moderated mediation effect for promotive voice (indirect effect = 0.14, 95% bias-corrected bootstrap CI [0.02, 0.28], excluding zero). Therefore, Hypothesis 5a was supported. In contrast, for prohibitive voice, the index of moderated mediation was not significant (indirect effect = −0.03, 95% bias-corrected bootstrap CI [−0.19, 0.12], including zero), so Hypothesis 5b was not supported.

**TABLE 3:** Results of conditional indirect effect analysis.

			**95%CI**		**Index of moderated mediation^b^**		
**Dependent variable**	**Sense of Impact**	**Estimate**	**LL**	**HL^a^**	**Index**	**Low**	**High**
Promotive voice	Low (−1 SD)	−0.27	−0.41	−0.16	0.14	0.02	0.28
	High (+1 SD)	−0.13	−0.26	−0.03			
Prohibitive voice	Low (−1 SD)	−0.17	−0.30	−0.05	−0.03	−0.19	0.12
	High (+1 SD)	−0.19	−0.36	−0.07			

*^a^LL, lower limit; HL, higher limit.*

*^b^95% confidence intervals of difference between high and low values of sense of impact.*

## Discussion

This research aimed to investigate how POP influences promotive and prohibitive voice, considering organizational embeddedness as a mediator and sense of impact as a moderator. In a three-wave survey utilizing the COR theoretical framework, we found that organizational embeddedness mediated the relationship between POP and promotive and prohibitive voice. Furthermore, we discovered that the positive relationship between organizational embeddedness and promotive voice was stronger for employees with a weak sense of impact. However, we did not find a similar effect for prohibitive voice. Additionally, sense of impact weakened the mediating effect of organizational embeddedness in the relationship between POP and promotive voice, whereas it did not have this effect with respect to prohibitive voice. These findings implied that organizational embeddedness transmits the negative effect of POP on voice behavior, and revealed that sense of impact to some extent can offset the negative effect of POP on promotive voice behavior. When employees perceive that they have a strong impact, they believe that they can influence decisions and even control the outcomes of voice behavior. This encourages employees to express their opinions. The results have further significant implications on employee voice behavior, POP, and COR theory.

It is worth noting that sense of impact’s moderating effect was significant for promotive voice but not for prohibitive voice. This may be due to the difference in resource demands between the two types of voice behavior. Promotive voice focuses on ideas to benefit the organization by changing the status quo (Van Dyne and LePine, 1998; Van Dyne et al., 2003). Its cooperative nature makes it more accepted by others and reduces employees’ risk of being misunderstood and retaliated against (Van Dyne et al., 2003). Therefore, employees need few resources to engage in promotive voice behavior. Prohibitive voice focuses on criticizing existing problems to prevent deteriorating conditions and future losses (Van Dyne et al., 2008; Liang et al., 2012). It can easily destroy interpersonal relationships and can cause employees to be viewed as troublemakers (Chamberlin et al., 2017; Ding et al., 2018). Therefore, employees need more resources to exhibit prohibitive voice. The plausible explanation may be that risk prevention requires many resources.

Due to the relatively few resources required, when employees have a strong sense of impact, their internal resources are adequate to stimulate promotive voice behavior; thus, the additional external resources provided by organizational embeddedness have a weak influence on such behavior. On the contrary, when employees have a weak sense of impact, their internal resources are not adequate to stimulate promotive voice behavior; thus, the external resources provided by organizational embeddedness play a significant role—that is, organizational embeddedness has a stronger influence on promotive voice. Therefore, sense of impact has a significant moderating effect on promotive voice. Based on COR theory, this seems to imply that for low-resource-demanding behaviors, the facilitative effects of internal and external resources may be substituted for each other.

For high-resource-demanding behaviors, such as prohibitive voice behavior, based on COR theory, internal and external resources are more likely to be in a complementary relationship, jointly contributing to the occurrence of high-resource-demanding behaviors. Thus, regardless of whether the employee’s sense of impact is strong or weak, the additional external resources provided by organizational embeddedness can still play a significant role alongside individuals’ own internal resources. Therefore, for prohibitive voice, sense of impact is not a significant moderator.

### Theoretical Implications

Our study has three theoretical implications. First, by introducing organizational embeddedness as a mediating variable, we have clarified the mechanism of POP’s effect on employee voice through the motivational theory of COR, which extends the existing research. Our research provides important evidence that POP, as a typical stress source, blocks many resources related to organizational embeddedness (e.g., relationships with others in the organization), thus inhibiting its development; in turn, this indirectly and negatively impacts promotive and prohibitive voice behaviors. As a proximal antecedent, organizational embeddedness motivates more employee voice behavior, which aligns with several prior studies (Ng and Feldman, 2013; Ng and Lucianetti, 2018; Tan et al., 2019). Previous studies have treated organizational embeddedness as an intermediary variable to convey the influence of leadership and positive context on performance and turnover (Ghosh et al., 2017; Jolly and Self, 2020; Siddique et al., 2020; Yang et al., 2020; Yu et al., 2020). Our finding enriches the nomological network of organizational embeddedness and extends the current literature on the POP-voice linkage.

Second, this research demonstrates that sense of impact is a boundary condition for the relationship between POP and promotive voice as mediated by organizational embeddedness. The positive relationship between organizational embeddedness and promotive voice is stronger for individuals with a weak sense of impact, and the mediating effect between POP and promotive voice through organizational embeddedness is also stronger for individuals with a weak sense of impact. Such knowledge helps us realize how internal resources play a role. Although organizational politics is inevitable (Ferris et al., 2019), we can increase internal resources to alleviate the negative effects of POP. This finding can help us better understand the effects of POP and organizational embeddedness on promotive voice.

Third, this research extends COR theory. By identifying sense of impact as a boundary condition and discussing the different interaction effects of external and internal resources on promotive and prohibitive voice behavior, we have gained a deeper understanding of the mechanisms of internal and external resources. For low-resource-demanding behaviors, if total resources reach a certain threshold, it is enough to stimulate promotive voice behavior. Therefore, when internal resources (sense of impact) are sufficient, the marginal effect of external resources (organizational embeddedness) is relatively small. For high-resource-demanding behaviors like prohibitive voice behavior, both kinds of resources are important. No matter how many or few internal resources are, external resources can play an important role. One could argue that for different resource-demanding behaviors, various individual resources may be complementary or act as substitutes (Hobfoll et al., 1990; Hobfoll, 2001). Our study is one of the few empirical studies to explore the relationship between types of resources, thus extending COR theory.

### Practical Implications

Our study provides some practical implications for managers to foster employee voice behavior. First, we found that POP adversely impacts promotive and prohibitive voice behavior. Organizations can reduce employees’ POP to improve their voice behavior. For example, managers should make the organization’s procedures more formalized, more transparent, and less ambiguous to guarantee that the distribution system is tied to tasks rather than individual preferences (Jarrett, 2017), as this is the only way employees can anticipate fair treatment if they give advice. Furthermore, managers should try to provide timely feedback to reduce employees’ perceptions of organizational politics (Rosen et al., 2006).

Second, considering that our research demonstrates that organizational embeddedness can increase voice behavior, organizations should reinforce employees’ embeddedness. For instance, organizations could design interactive and interdependent work and could host networking activities to build relationships (Mossholder et al., 2011; Collins and Mossholder, 2017). Organizations could also provide training to increase employees’ qualifications for their jobs and facilitate employees’ career development to increase fit (Tan et al., 2019). Finally, organizations could appropriately increase benefits (e.g., income or status) to elevate the cost of leaving (i.e., sacrifice).

Third, it is important to note that the internal resource of sense of impact can motivate employees’ voice behavior, organizations should increase internal resources like psychological well-being, leadership skills, sense of impact, etc., to mitigate the negative effects of POP to some extent. For example, the organization can add some training on leadership skill, positive and optimistic mindset; further, when managers make decisions, they can solicit more opinions from employees to make them feel that their opinions are valued, thus increasing their sense of impact (Tangirala and Ramanujam, 2008, 2012).

### Limitations and Directions for Further Research

As in all research, we must acknowledge the limitations of our study and then provide some future directions to improve related research. First, we cannot clarify causal relationships because of the cross-sectional design, although we have used a time-lagged survey to compensate for this design limitation. Thus, future studies could implement experimental designs to provide more solid evidence for a causal effect of POP on voice behavior. Second, the current study did not consider the boundary conditions of the role of POP on organizational embeddedness, leader-member exchange (LMX) may have a moderating effect between them, and in-group and out-group members may have different sensitivities and reactions to POP (Rosen et al., 2011; Chhetri et al., 2014), thus influencing the effect of POP on organizational embeddedness and, in turn, affects employee voice behavior. Future studies can further refine the research model by considering LMX as a moderating variable in the first stage. Finally, as this study was conducted within a Chinese cultural context, the relationships among the variables in our model may not necessarily hold in other cultural contexts. Future research should consider a more diverse sample, or cross-cultural studies could explore the generalizability of our findings.

## Conclusion

Our study explains the effect of POP on employee voice from a COR perspective and finds that organizational embeddedness transmits the negative effects of POP to employee voice behavior. We also find that organizational embeddedness has a stronger effect on promotive voice for employees with a lower sense of impact, whereas there is no significant difference in the effect of organizational embeddedness on prohibitive voice regardless of whether employees have a high or low sense of impact. In addition, we also find that POP has a stronger effect on promotive voice through organizational embeddedness for employees with a lower sense of impact, while there is no significant difference in the effect of POP on prohibitive voice, regardless of whether employees’ sense of impact was high or low. In conclusion, this research enriches our understanding of POP, voice behavior, and conservation of resources theory.

## Data Availability Statement

The raw data supporting the conclusions of this article will be made available by the authors, without undue reservation.

## Ethics Statement

Ethical review and approval was not required for the study on human participants in accordance with the local legislation and institutional requirements. Written informed consent for participation was not required for this study in accordance with the national legislation and the institutional requirements.

## Author Contributions

QL: conceptualization, methodology, software, formal analysis, and original draft preparation. HZ: writing, reviewing, editing of the manuscript, project administration, and funding acquisition. XS: methodology and software. All authors contributed to the article and approved the submitted version.

## Conflict of Interest

The authors declare that the research was conducted in the absence of any commercial or financial relationships that could be construed as a potential conflict of interest.

## Publisher’s Note

All claims expressed in this article are solely those of the authors and do not necessarily represent those of their affiliated organizations, or those of the publisher, the editors and the reviewers. Any product that may be evaluated in this article, or claim that may be made by its manufacturer, is not guaranteed or endorsed by the publisher.

## Appendix

Measures of Core Constructs

### POP

1. Favoritism rather than merit determines who gets ahead around here.
2. Rewards come only to those who work hard in this organization. (R).
3. There is a group of people in my department who always get things their way because no one wants to challenge them.
4. People in this organization attempt to build themselves up by tearing others down.
5. I have seen changes made in policies here that only serve the purposes of a few individuals, not the work unit or the organization.
6. People here usually don’t speak up for fear of retaliation by others.

### Organizational Embeddedness

1. I feel compatible with my organization.
2. I feel a strong link to my organization.
3. I would sacrifice a lot if I left this job.

### Sense of Impact

1. My impact on what happens in my department is large.
2. I have a great deal of control over what happens in my department.
3. I have significant influence over what happens in my department.

### Promotive Voice

1. I proactively develop and make suggestions for issues that may influence the unit.
2. I proactively suggest new projects which are beneficial to the work unit.
3. I raise suggestions to improve the unit’s working procedure.
4. I proactively voice out constructive suggestions that help the unit reach its goals.
5. I make constructive suggestions to improve the unit’s operation.

### Prohibitive Voice

1. I speak up honestly with problems that might cause serious loss to the work unit, even when/though dissenting opinions exist.
2. I advise other colleagues against undesirable behaviors that would hamper job performance.
3. I dare to voice out opinions on things that might affect efficiency in the work unit, even if that would embarrass others.
4. I dare to point out problems when they appear in the unit, even if that would hamper relationships with other colleagues.
5. I proactively report coordination problems in the workplace to the management.

### Zhongyong

1. When dealing with colleagues, it is not enough to be reasonable, but also to be compassionate.
2. Everything has limitations, so it is not very good to exceed them.
3. When making decisions, I will adjust myself for the overall harmony.
4. I will consider other people’s ideas and practices in order to be consistent with them.
5. I will consider all possible situations when I do things.
6. I will find a compromise or balance between different opinions.
